# Urinary potassium excretion and mortality risk in community-dwelling individuals with and without obesity

**DOI:** 10.1093/ajcn/nqac137

**Published:** 2022-05-17

**Authors:** Stanley M H Yeung, Anne Nooteboom, Ewout J Hoorn, Joris I Rotmans, Liffert Vogt, Rudolf A de Boer, Ron T Gansevoort, Gerjan Navis, Stephan J L Bakker, Martin H De Borst

**Affiliations:** Department of Internal Medicine, Division of Nephrology, University of Groningen, University Medical Center Groningen, Groningen, The Netherlands; Department of Internal Medicine, Division of Nephrology, University of Groningen, University Medical Center Groningen, Groningen, The Netherlands; Department of Internal Medicine, Division of Nephrology & Transplantation, Erasmus Medical Center, University Medical Center Rotterdam, Rotterdam, The Netherlands; Department of Internal Medicine, Leiden University Medical Center, Leiden, The Netherlands; Department of Internal Medicine, Section of Nephrology, Amsterdam Cardiovascular Sciences, Amsterdam University Medical Centers, University of Amsterdam, Amsterdam, The Netherlands; Department of Cardiology, University of Groningen, University Medical Center Groningen, Groningen, The Netherlands; Department of Internal Medicine, Division of Nephrology, University of Groningen, University Medical Center Groningen, Groningen, The Netherlands; Department of Internal Medicine, Division of Nephrology, University of Groningen, University Medical Center Groningen, Groningen, The Netherlands; Department of Internal Medicine, Division of Nephrology, University of Groningen, University Medical Center Groningen, Groningen, The Netherlands; Department of Internal Medicine, Division of Nephrology, University of Groningen, University Medical Center Groningen, Groningen, The Netherlands

**Keywords:** potassium intake, nutrition, epidemiology, general population, body dimension, mortality

## Abstract

**Background:**

Potassium intake has been shown to be inversely associated with blood pressure and premature mortality. Previous studies have suggested that the association between potassium intake and blood pressure is modified by obesity, but whether obesity similarly influences the association between potassium intake and mortality is unclear.

**Objectives:**

We investigated whether potassium intake, reflected by 24-h urinary excretion, is associated with all-cause mortality, and explored potential effect modification by obesity.

**Methods:**

We performed a prospective cohort study in community-dwelling individuals. The association between urinary potassium excretion and all-cause mortality was investigated by using multivariable Cox regression. We performed multiplicative interaction analysis and subgroup analyses according to BMI and waist circumference.

**Results:**

In 8533 individuals (50% male), the mean age was 50 ± 13 y, mean urinary potassium excretion was 71 ± 21 mmol/24 h, median BMI (in kg/m^2^) was 25.6 (IQR: 23.1, 28.4) and mean waist circumference was 89 ± 13 cm. During median follow-up of 18.4 (IQR: 13.5, 18.8) y, 1663 participants died. Low urinary potassium excretion (first compared with third sex-specific quintile) was associated with an increased mortality risk (fully adjusted HR: 1.38; 95% CI: 1.18, 1.61), *P* < 0.001, irrespective of body dimensions (HR range for all body dimensions: 1.36–1.70, all *P* < 0.05). High urinary potassium excretion (fifth compared with third quintile) was associated with increased mortality risk in participants with obesity (BMI ≥30; HR: 1.52; CI: 1.00, 2.30), but not in participants without obesity (BMI: <25; HR: 0.89; 95% CI: 0.62, 1.26; *P*-interaction = 0.001).

**Conclusions:**

Low potassium intake was associated with increased mortality risk in community-dwelling individuals. In individuals with obesity, high potassium intake was also associated with increased mortality risk.

## Introduction

Low urinary potassium excretion is independently associated with a higher risk of cardiovascular disease (CVD) (1) and premature mortality (2,3). Intervention studies have demonstrated that potassium supplementation decreases blood pressure (4). A recent cluster randomized trial demonstrated that a salt substitute, partly replacing sodium with potassium salt, reduced mortality risk in individuals with a history of stroke or age >60 y and high blood pressure (5). Possible explanations for the beneficial effects of potassium supplementation include potassium-induced natriuresis through inhibition of the sodium-chloride cotransporter and direct vascular effects (6).

Previous studies have demonstrated that individuals with obesity are salt sensitive, as reflected by a stronger effect of sodium loading on blood pressure (7,8). Because potassium-induced natriuresis is considered a main mechanism by which potassium lowers blood pressure, it has also been hypothesized that individuals with obesity are more sensitive to potassium-induced blood pressure–lowering effects. Indeed, a previous study demonstrated that waist circumference was associated with a stronger blood pressure response to potassium supplementation (9). In line with these findings, a study in Chinese adults reported that the association between urinary potassium excretion and incident hypertension was modified by BMI (10).

Whether the association between potassium intake and mortality is modified by obesity is unknown. Therefore, the aim of the current study was to assess whether urinary potassium excretion is associated with all-cause mortality in a population-based cohort, and whether this association is influenced by BMI or waist circumference.

## Methods

### Study Population

The Prevention of Renal and Vascular End-Stage Disease (PREVEND) study is a prospective cohort study investigating the natural course of increased amounts of urinary albumin excretion and its relation to renal disease and CVD in a large cohort drawn from the general population. Study details have been described extensively elsewhere (11). In brief, from 1997 and 1998, all inhabitants of Groningen, the Netherlands, aged 28 to 75 y, were sent a questionnaire and a vial to collect a first-morning void urine sample. Pregnant female subjects and insulin users were excluded, since the original aim of PREVEND was to study the relation between albuminuria and outcomes in individuals without type 1 diabetes. Participants with urinary albumin excretion >10 mg/L in a spot morning urine sample were invited (*n* = 7768), of whom 6000 participants were enrolled. In addition, persons with a lower albumin excretion were randomly invited (*n* = 3395), of whom 2592 were enrolled eventually. These 8592 participants formed the PREVEND cohort and completed the baseline examination, which consisted of 2 visits within 2 wk. In the follow-up period, a portion of the participants completed the subsequent examinations between 2001 and 2008. In the current study, we included only participants who had available 24-h measurement results for urinary sodium and potassium excretion and who completed the baseline examination (*n* = 8533) (**Supplemental Figure 1**). The PREVEND study has been approved by the medical ethics committee of the University Medical Center Groningen and was performed in accordance with Declaration of Helsinki guidelines. Written informed consent was obtained from all participants.

### Data Collection

Each examination consisted of 2 visits to the outpatient clinic, separated by 3 wk. At baseline, participants completed a questionnaire on demographics, history of CVD (defined as self-reported myocardial infarction, percutaneous transluminal coronary angioplasty, coronary artery bypass graft, or cerebrovascular accident), type 2 diabetes (defined as self-reported type 2 diabetes, use of oral hypoglycemic drugs, or glucose measurement), smoking habits, alcohol usage, education [categorized into low (primary education up to those completing intermediate vocational education), average (higher secondary education), and high (higher vocational education and university)] and medication use. Information on medication use at baseline was amended with data acquired from IADB.nl, a database comprising pharmacy dispensing data from all community pharmacies in Groningen.

The proportion of participants who had received at ≥1 prescription during the 6 mo before screening is presented as prevalent drug use at baseline. Use of lipid-lowering, oral hypoglycemic, and antihypertensive drugs was recorded. Antihypertensive drugs were defined as angiotensin-converting enzyme inhibitors, angiotensin receptor blockers, thiazide diuretics, loop diuretics, beta-blockers, calcium channel blockers, potassium sparing diuretics, or other antihypertensives. In the last week before the second visit (i.e., visit 2/2 of the baseline examination), participants collected 2 consecutive 24-h urine samples. The collected urine was stored cold at 4°C for a maximum of 4 d. After the collected urine was received, urine specimens were stored at −20°C. We used the average of both 24-h urine collections that were part of the baseline examination in our analyses. Furthermore, fasting blood samples were taken and stored at −80°C. During the visit, blood pressure was assessed on the right arm in the supine position every minute for 10 and 8 min, respectively, by an automatic Dinamap XL Model 9300 series device. The mean of the last 2 recordings from each visit was used. The mean arterial pressure was calculated using these recordings. BMI was calculated using weight (kilograms) divided by height squared (square meters). Minimum waist circumference was measured on bare skin at the natural indentation between the 10th rib and the iliac crest. When there was no indentation, we measured it in the middle between navel and rib cage. Smoking status was categorized as current or not. Alcohol intake was categorized as current or no alcohol consumption. The primary endpoint was all-cause mortality. The outpatient program of the hospital uses a continuous surveillance system by the municipal registration of death to ensure up-to-date information on patient status (alive or deceased).

### Laboratory Measurements

The presence of potassium, sodium, urea, and creatinine in urine samples was determined with a MEGA clinical chemistry analyzer (Merck). Potassium, and sodium were determined by indirect potentiometry, and urea was determined by a photometric test with the urease-GIDH method. Estimated glomerular filtration rate (eGFR) was calculated based on the combined creatinine and cystatin C Chronic Kidney Disease-Epidemiology Collaboration equation (12). Serum and urinary creatinine, plasma total cholesterol, and triglycerides were determined from fasting blood samples by using Kodak Ektachem dry chemistry (Eastman Kodak). Plasma HDL cholesterol was measured with a homogeneous method (direct HDL, Aeroset TM System, Abbott Laboratories). Plasma renin concentration was measured using an automated sandwich immunochemiluminescent assay (LIAISON®, Diasorin). Serum aldosterone concentration was measured using a competition-based radioimmunoassay (ALDOCTK-2, Diasorin). Serum cystatin C concentrations were measured by a Gentian Cystatin C Immunoassay (Gentian AS) on a modular analyzer (Roche Diagnostics). Urinary albumin concentration was determined by nephelometry (Dade Behring Diagnostic). Urinary albumin-to-creatinine ratio (ACR) was calculated by dividing the urinary albumin concentration (mg/L) by the urinary creatinine concentration (mmol/L).

### Statistical Analyses

Baseline characteristics are presented according to quintiles of urinary potassium excretion. Continuous data are presented as mean ±  SD or as median (IQR) in case of nonnormal distribution. Categorical data are presented as percentiles. Nonnormally distributed variables were transformed before being used in subsequent analyses if needed. We aimed to identify correlates of urinary potassium excretion by using a chi-square test, 1-way ANOVA, or Kruskal Wallis test for nominal, normally distributed, and nonhomogeneity or nonnormally distributed data, respectively. In order to avoid potential bias from exclusion of patients with missing values, we used multiple imputation (fully conditional specification (Markov Chain Monte-Carlo) to obtain 5 imputed datasets (13). Rubin's rules were used to obtain pooled estimates of the regression coefficients and their SEs across imputed datasets (14).

We used Cox proportional multivariable hazards regression analyses to investigate the associations of urinary potassium excretion, in sex-specific quintiles, with all-cause mortality. The third (middle) quintile was defined as the reference category. Survival time was defined from baseline until date of death, or 1 January 2017 (end of follow-up). We adjusted for confounders and we built the fully adjusted model as follows: sex-specific quintiles of urinary potassium excretion adjusted for age, BMI (indirect measurement of caloric intake), eGFR, ACR (kidney function), risk factors for mortality, history of CVD, type 2 diabetes, education level, alcohol consumption, smoking, triglyceride-HDL ratio, urinary creatinine excretion as a marker for muscle mass (15), urinary urea excretion as a marker of protein intake, urinary sodium excretion, and use of oral hypoglycemic, antihypertensive, and cholesterol lowering drugs. In addition, we adjusted for aldosterone, renin, and blood pressure to find potential mediation effects of these variables. Nonlinearity was tested by using a likelihood ratio test, comparing nested models with linear or linear and cubic terms. We used restricted cubic spline with 3 knots (5th, 50th, and 95th percentile) to visualize the continuous association between urinary potassium excretion and risk of all-cause mortality by fitting the Cox regression analysis of the final model. Furthermore, we investigated whether urinary potassium excretion was associated with CVD and non-CVD mortality.

We analyzed potential effect modification by BMI and waist circumference by fitting models containing both main effects and their cross-products terms in the all-cause mortality analyses. We categorized participants according to 3 BMI groups (<25, 25–30, and ≥30, measured in kg/m^2^) or waist circumference (<82, 82−94, ≥94 cm) and quintiles of urinary potassium excretion. The groups with BMI <25 and waist circumference 82–94 cm, respectively, were defined as the reference categories.

To examine the robustness of the main findings, we performed sensitivity analyses by excluding subjects with potential inadequacy of 24-h collection by comparing the actual 24-h creatinine excretion with creatinine excretion as predicted by body dimensions, age, and sex (16). In addition, to further increase the robustness of the main findings, we performed sensitivity analyses by excluding participants with history of CVD or malignancy and participants who developed chronic kidney disease (defined as a combination of reaching an eGFR of <60 mL/min per 1.73 m^2^ or a urinary albumin excretion of >30 mg/24 h de novo, or both), cardiovascular event, or new-onset malignancy. Cardiovascular event was coded according to the *International Classification of Diseases, Ninth Revision*, and consisted of the combined incidence of fatal and nonfatal events of ischemic heart disease, stroke, and vascular interventions such as percutaneous transluminal angioplasty or bypass grafting of aorta and peripheral vessels. Ischemic heart disease events were defined as acute myocardial infarction (code 410), acute and subacute ischemic heart disease (code 411), coronary artery bypass grafting (code 414), or percutaneous transluminal coronary angioplasty (code 36.0). Stroke events were defined as subarachnoid hemorrhage (code 430), intracerebral hemorrhage (code 431), other intracranial hemorrhage (code 432), or occlusion or stenosis of the precerebral (code 433) or cerebral (code 434) arteries. In addition, strokes were subclassified into hemorrhagic strokes (codes 430–431), ischemic strokes (codes 433–434), or unspecified strokes (code 432). Data on fatal CVD were received through the municipal register. Cause of death was obtained by linking the number of the death certificate to the primary cause of death as coded by the Dutch Central Bureau of Statistics. New-onset malignancy cases (included all malignancies, except nonmelanoma skin cancers) were provided through the nationwide network and registry of histopathology and cytopathology in the Netherlands (PALGA) (17) and were reviewed and adjudicated by an independent committee. To analyze the long-term effect of potassium intake on the outcome, sensitivity analysis was done by excluding participants who died or were lost to follow-up ≤1 y after baseline. As the last step, sensitivity analyses were done by adjustment for renin–angiotensin–aldosterone system (RAAS) inhibitor or diuretic use in the follow-up and by repeating the main analysis while excluding participants who were using RAAS inhibitors or diuretics.

All statistical analyses were performed with SPSS software version 23.0 for Windows (IBM), and R version 4.0.1 (http://cran.r-project.org/). In all analyses, a 2-sided *P*-value < 0.05 was considered significant.

## Results

### Patient Characteristics

Data were analyzed from 8533 individuals (50% male) with a mean age of 50 ± 13 y. Mean urinary potassium excretion was 71 ± 21 mmol/24 h, median BMI was 25.6 (IQR 23.1, 28.4), and mean waist circumference 89 ± 13 cm. Baseline characteristics according to sex-specific quintiles of urinary potassium excretion are displayed in Table 1. Urinary potassium excretion correlated with urinary sodium excretion (*r* = 0.45, *P* < 0.001), and individuals in the highest urinary potassium excretion quintiles also had the highest urinary sodium excretion [median 163 (IQR 129–202) mmol/24 h, *P-*trend < 0.001), as well as higher urinary urea excretion, suggesting higher protein intake (Table 1). Participants with a higher urinary potassium excretion had a slightly higher BMI, while those with a lower urinary potassium excretion had higher total cholesterol, triglycerides, and triglyceride/HDL ratio and lower HDL cholesterol. Higher serum aldosterone concentrations were seen in participants with higher urinary potassium excretion, but plasma renin was not different. The number of participants with missing data for each variable is presented in **Supplemental Table 1**.

**TABLE 1:** Baseline characteristics according to sex specific quintiles of urinary potassium excretion in 8533 participants of PREVEND^1^

	Quintiles of total urinary potassium excretion						
	Total	I	II	III	IV	V	*P*-trend^2^
Sex-specific quintiles, mmol/24 h							
Male (*n* = 4301)	77 ± 22	<59	59–70	70–81	81–94	>94	
Female (*n* = 4291)	66 ± 19	<50	50–60	60–69	69–81	>81	
Urinary potassium excretion, mmol/24 h	71 ± 21	44 ± 9	60 ± 6	70 ± 6	80 ± 7	101 ± 15	—
Age, y	50 ± 13	52 ± 13	51 ± 13	50 ± 13	49 ± 12	47 ± 11	<0.001
BMI	25.6 (23.1–28.4)	25.4 (22.8–28.1)	25.6 (23.2–28.2)	25.7 (23.2–28.4)	25.5 (23.1–28.4)	25.7 (23.4–28.9)	0.004
Waist circumference, cm	89 ± 13	89 ± 13	89 ± 13	88 ± 13	89 ± 13	89 ± 13	0.15
Systolic blood pressure, mm Hg	126 (114–141)	127 (114–143)	127 (114–142)	125 (114–140)	125 (114–140)	125 (113–139)	0.006
Diastolic blood pressure, mm Hg	73 (67–80)	74 (67–81)	74 (68–81)	73 (67–80)	73 (67–79)	72 (67–79)	<0.001
Antihypertensive drugs	1164 (14)	290 (17)	255 (15)	249 (15)	190 (12)	180 (11)	<0.001
ACE-inhibitors/ARB	404 (6)	98 (7)	86 (6)	88 (6)	66 (5)	66 (5)	0.09
Thiazide diuretics	201 (3)	49 (3)	42 (3)	38 (3)	23 (2)	49 (4)	0.02
Loop diuretics	66 (0.9)	19 (1)	11 (0.8)	11 (0.7)	14 (1)	11 (0.8)	0.48
Potassium-sparing diuretics	14 (0.2)	3 (0.2)	3 (0.2)	1 (0.1)	4 (0.3)	3 (0.2)	0.77
Lipid-lowering drugs	346 (5)	92 (6)	77 (6)	87 (6)	44 (3)	46 (3)	<0.001
Oral hypoglycemic drugs	119 (2)	28 (2)	24 (2)	24 (2)	22 (2)	21 (2)	0.94
Education level							<0.001
Low	3674 (43)	880 (52)	824 (49)	719 (42)	678 (40)	573 (34)	
Middle	1943 (23)	386 (23)	399 (24)	392 (23)	384 (23)	382 (23)	
High	2874 (34)	429 (25)	474 (28)	588 (35)	640 (38)	743 (44)	
Smoking status, ever	2912 (34)	679 (40)	607 (36)	589 (35)	527 (31)	510 (31)	<0.001
Alcohol consumption, yes	6328 (75)	1114 (66)	1246 (73)	1301 (76)	1309 (77)	1358 (80)	<0.001
CVD, yes	449 (5)	141 (8)	96 (6)	93 (5)	65 (4)	54 (3)	<0.001
Type 2 diabetes, yes	245 (3)	51 (3)	52 (3)	44 (3)	54 (3)	44 (3)	0.76
History of malignancy, yes	134 (2)	28 (2)	33 (2)	26 (2)	24 (1)	23 (1)	0.68
Plasma creatinine, µmol/L	70.7 (61.0–80.4)	69.6 (61.0–80.4)	70.7 (61.0–80.4)	70.7 (62.1–80.4)	70.7 (62.1–80.4)	70.7 (62.1–80.4)	0.55
eGFR, mL/min·1.73m^2^	93.4 ± 21.3	90.9 ± 22.5	92.2 ± 21.9	93.1 ± 21.3	94.3 ± 20.6	96.3 ± 19.6	<0.001
Stage 1 (eGFR ≥90)	4455 (56)	803 (51)	860 (54)	875 (55)	929 (58)	988 (62)	
Stage 2 (eGFR ≥60 and <90)	3027 (38)	637 (41)	605 (38)	633 (40)	590 (37)	562 (35)	
Stage 3 (eGFR ≥30 and <60)	459 (6)	126 (8)	121 (8)	88 (6)	78 (5)	46 (3)	
Stage 4 (eGFR ≥15 and <30)	19 (0.2)	6 (0.4)	6 (0.4)	4 (0.3)	3 (0.2)	0 (0)	
Stage 5 (eGFR <15)	2 (0)	2 (0.1)	0 (0)	0 (0)	0 (0)	0 (0)	
Plasma albumin, g/L	45.8 ± 2.7	45.6 ± 2.8	45.6 ± 2.8	45.9 ± 2.7	45.9 ± 2.6	45.9 ± 2.6	<0.001
Plasma potassium, mmol/L	4.4 ± 0.7	4.3 ± 0.4	4.4 ± 0.6	4.4 ± 0.7	4.4 ± 0.8	4.4 ± 0.6	<0.001
Plasma sodium, mmol/L	142 ± 2.3	142 ± 2.4	142 ± 2.5	142 ± 2.4	142 ± 2.3	142 ± 2.0	0.007
Serum aldosterone, pmol/L	118 (93–153)	116 (89–155)	120 (94–157)	120 (93–155)	117 (92–157)	123 (96–160)	0.001
Plasma renin, ng/L	18 (1–29)	18 (10–29)	19 (11–30)	18 (11–29)	19 (11–29)	18 (12–28)	0.56
Total cholesterol, mmol/L	5.6 (4.9–6.3)	5.6 (4.9–6.4)	5.6 (4.9–6.4)	5.6 (4.9–6.4)	5.5 (4.9–6.3)	5.4 (4.8–6.2)	<0.001
HDL cholesterol, mmol/L	1.32 ± 0.40	1.30 ± 0.39	1.31 ± 0.40	1.32 ± 0.39	1.33 ± 0.40	1.34 ± 0.41	0.02
Triglycerides, mmol/L	1.16 (0.85–1.68)	1.22 (0.90–1.75)	1.19 (0.85–1.71)	1.18 (0.86–1.69)	1.11 (0.82–1.66)	1.11 (0.82–1.63)	<0.001
Triglyceride/HDL ratio^3^	2.10 (1.30–3.51)	2.27 (1.41–3.73)	2.15 (1.35–3.56)	2.12 (1.31–3.47)	2.00 (1.23–3.42)	1.95 (1.23–3.33)	<0.001
Urinary sodium excretion, mmol/24 h	136 (106–170)	109 (82–138)	128 (101–157)	139 (109–171)	145 (118–178)	163 (129–202)	<0.001
Sodium/potassium ratio	2.0 (1.6–2.5)	2.5 (1.9–3.0)	2.1 (1.7–2.6)	2.0 (1.6–2.4)	1.8 (1.5–2.2)	1.6 (1.3–2.0)	<0.001
Urinary urea excretion, mmol/24 h	354 ± 106	269 ± 81	323 ± 82	360 ± 88	383 ± 93	436 ± 104	<0.001
Urinary creatinine excretion, mmol/24 h	12.3 ± 3.5	10.3 ± 3.1	11.6 ± 3.0	12.4 ± 3.1	13.0 ± 3.5	14.1 ± 3.8	<0.001
ACR, mg/g in 24-h urine	0.80 (0.54–1.49)	0.86 (0.56–1.72)	0.83 (0.55–1.56)	0.79 (0.53–1.43)	0.78 (0.54–1.43)	0.74 (0.52–1.39)	<0.001

1 Data are presented as *n* (%), means ± SDs, or medians (IQRs) for nominal, normally distributed, and non-normally distributed data, respectively. ACE, angiotensin-converting enzyme; ACR, albumin-to-creatinine ratio; ARB, angiotensin receptor blocker; CRP, C-reactive protein; CVD, cardiovascular disease; eGFR, estimated glomerular filtration rate; PTH, parathyroid hormone.

2 The *P*-value represents the *P* for trend in the chi-square test, 1-way-ANOVA, or Kruskal–Wallis test for nominal, normally distributed and nonhomogeneity or nonnormally distributed data, respectively.

3 Triglyceride/HDL ratio was calculated using mg/dL units.

### Associations with Mortality

**FIGURE 1:**
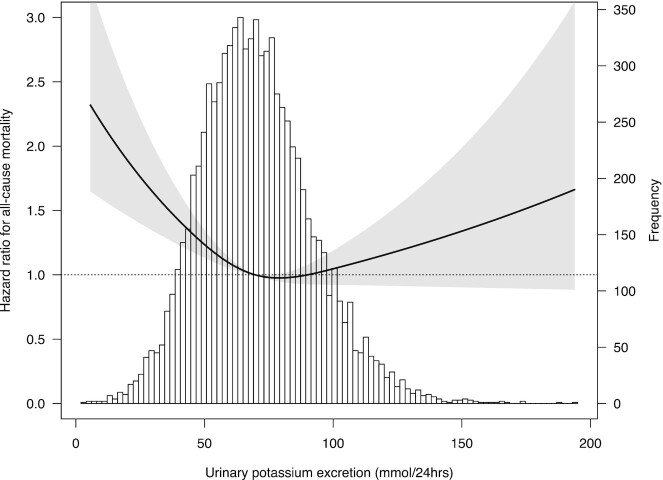
Associations between urinary potassium excretion and all-cause mortality in 8533 participants. Data were fit by a Cox proportional hazards regression model based on restricted cubic splines (5th, 50th, and 95th percentile knots) and adjusted for age, sex, eGFR, BMI, urinary albumin-to-creatinine excretion, type 2 diabetes, education level, alcohol consumption, smoking, history of CVD, triglyceride HDL ratio, urinary creatinine excretion, urea excretion, sodium excretion, antihypertensive, oral hypoglycemic and lipid lowering drugs. The gray area represents the 95% CI. Linear *P* = 0.003, nonlinear *P* < 0.001. CVD, cardiovascular disease; eGFR, estimated glomerular filtration rate.

During a median follow-up of 18.4 y (IQR: 13.5, 18.8), 1663 participants died (19%), and the mortality rate was distributed according to the sex-specific quintiles as follows: quintile I: 477 (28%); II: 364 (21%); III: 313 (18%); IV: 280 (16%); and V: 222 (13%). Upon Cox proportional hazards regression analysis, participants in the lowest sex-specific quintile of urinary potassium excretion had a significantly increased risk of all-cause mortality compared with participants the third quintile (fully adjusted HR: 1.38; 95% CI: 1.18, 1.61; *P* < 0.001, Table 2 and Figure 1). The HR of the highest sex-specific urinary potassium excretion quintile was similar to the reference (third) quintile (HR: 1.02; 95% CI: 0.85, 1.22). Further adjustment for serum aldosterone, plasma renin, and systolic and diastolic blood pressure did not materially change the results (**Supplemental Table 2**). Of all mortality cases, 441 were related to CVD and 1222 were not related to CVD. Participants in the lowest sex-specific quintile of urinary potassium excretion showed a trend in increased risk of CVD mortality compared with participants in the third quintile (fully adjusted HR: 1.33; 95% CI: 0.99, 1.78; *P =* 0.06, **Supplemental Table 3**). Furthermore, participants in the lowest sex-specific quintile of urinary potassium excretion had a significantly increased risk of non-CVD mortality compared with the third quintile (fully adjusted HR: 1.40; 95% CI: 1.17, 1.68; *P <* 0.001, Supplemental Table 3).

**TABLE 2:** Association of urinary potassium excretion with risk of all-cause mortality in 8533 subjects of PREVEND^1^

	Quintiles of urinary potassium excretion, mmol/24 h				
	I	II	III	IV	V
Sex-specific values, mmol/24 h					
Male (*n* = 4301)	<59	59–70	70–81	81–94	>94
Female (*n* = 4291)	<50	50–60	60–69	69–81	>81
HR adjusted for age	1.43 (1.24, 1.65)***	1.12 (0.96, 1.30)	1.0 (ref)	0.94 (0.80, 1.10)	0.98 (0.82, 1.16)
Fully adjusted HR^2^	1.38 (1.18, 1.61)***	1.13 (0.97, 1.32)	1.0 (ref)	0.99 (0.84, 1.16)	1.02 (0.85, 1.22)

1 Data are presented as HRs (95% CIs) unless otherwise indicated. *P*-values are shown as: *≤0.05, **≤0.01, ***<0.001. ACR, albumin-to-creatinine ratio; eGFR, estimated glomerular filtration rate; PREVEND, Prevention of Renal and Vascular End-Stage Disease, ref = reference.

2 Model additionally adjusted for BMI, eGFR, urinary ACR, type 2 diabetes, education level, alcohol consumption, smoking, history of cardiovascular disease, triglyceride/HDL ratio, urinary creatinine, urea, sodium excretion, and antihypertensive, oral hypoglycemic, and lipid lowering drugs.

### Interaction with Body Dimensions

**FIGURE 2:**
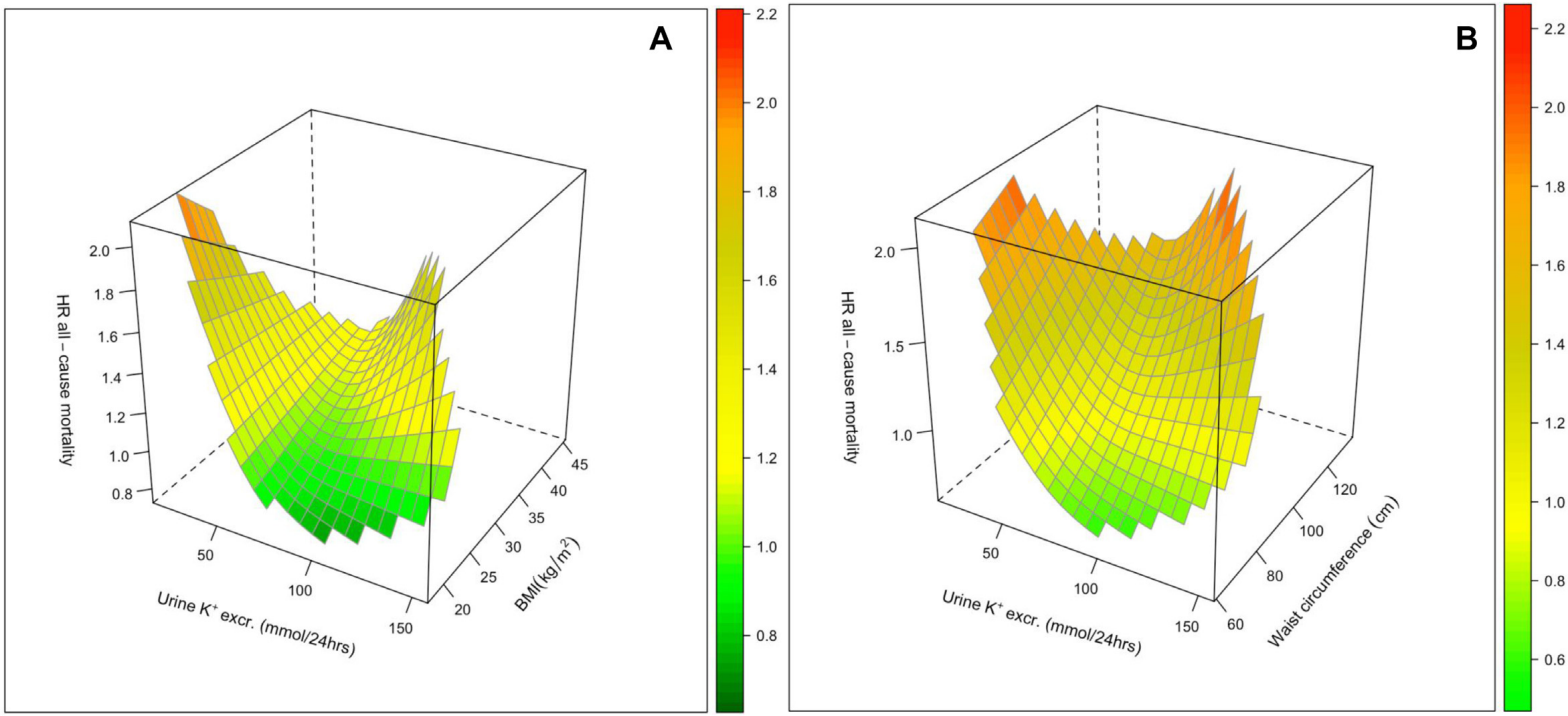
3D plots depicting the multivariable adjusted association between urinary potassium excretion (BMI, A; Waist circumference, B) and all-cause mortality in 8533 participants. Cox proportional hazards regression model was adjusted for age, sex, eGFR, BMI, urinary albumin-to-creatinine excretion, type 2 diabetes, education level, alcohol consumption, smoking, history of CVD, triglyceride HDL ratio, urinary creatinine excretion, urea excretion, sodium excretion, antihypertensive, oral hypoglycemic and lipid lowering drugs. CVD, cardiovascular disease; eGFR, estimated glomerular filtration rate.

Low urinary potassium excretion was associated with a higher risk of all-cause mortality in all BMI and waist circumference subgroups (Table 3, Figure 2A and B). Furthermore, high urinary potassium excretion was associated with a higher mortality risk in individuals with a BMI ≥30 or a waist circumference ≥94 cm, but not in those in the intermediate or lower BMI or waist circumference categories (*P*-interaction < 0.01; Table 3 and Figure 2A and B). Further adjustment for serum aldosterone, plasma renin, and systolic and diastolic blood pressure did not materially change the results in the individuals with highest BMI and waist circumference (**Supplemental Table 4**). Urinary sodium excretion increased over quintiles of potassium excretion in participants with obesity (**Supplemental Table 5**), and only 9% of participants with obesity in the highest potassium quintile had a sodium excretion <120 mmol/24 h.

**TABLE 3:** Association of urinary potassium excretion with all-cause mortality in body dimensions subgroups^1^

Sex-specific quintiles of urinary potassium excretion, mmol/24 h					
I	II	III	IV	V	*P*-interaction^2^
BMI <25					
165/738	102/740	90/739	79/740	48/738	
1.70 (1.31, 2.21)***	1.10 (0.83, 1.47)	1.0 (ref.)	1.17 (0.86, 1.59)	0.89 (0.62, 1.26)	0.001
BMI 25–30					
229/700	198/702	154/701	132/701	109/701	
1.36 (1.04, 1.79)*	1.34 (1.02, 1.76)*	1.11 (0.84, 1.47)	0.90 (0.67, 1.20)	1.11 (0.82, 1.51)	
BMI ≥30					
83/266	72/267	68/267	63/268	64/265	
1.47 (1.00, 2.16)*	1.35 (0.90, 2.02)	1.19 (0.79, 1.78)	1.11 (0.74, 1.67)	1.52 (1.00, 2.30)*	
Waist circumference <82 cm					
91/576	49/577	45/577	42/577	28/577	
1.62 (1.19–2.23)**	0.89 (0.62, 1.28)	0.84 (0.56, 1.21)	1.08 (0.74, 1.57)	0.90 (0.58, 1.40)	0.002
Waist circumference 82–94 cm					
148/541	112/541	85/542	75/541	52/541	
1.42 (1.08, 1.86)*	1.22 (0.92, 1.62)	1.0 (ref.)	1.01 (0.74, 1.38)	0.91 (0.64, 1.28)	
Waist circumference ≥94 cm					
236/588	208/591	190/586	150/589	142/589	
1.50 (1.16, 1.94)**	1.46 (1.13, 1.90)**	1.35 (1.04, 1.76)*	1.14 (0.86, 1.50)	1.36 (1.02, 1.80)*	

1 Data are presented as *n* events/*n* participants or HRs (95% CIs) unless otherwise indicated. ACR, albumin-to-creatinine ratio; eGFR, estimated glomerular filtration rate.

2 *P*-values shown as: * ≤0.05, ** ≤ 0.01, *** <0.001. Multivariable adjusted Cox regression models adjusted for age, BMI, eGFR, urinary ACR, type 2 diabetes, education level, alcohol consumption, smoking, history of cardiovascular disease, triglyceride HDL ratio, urinary creatinine, urea, sodium excretion, and antihypertensive, oral hypoglycemic, and lipid-lowering drugs.

### Sensitivity Analyses

In sensitivity analyses, we excluded participants with potentially inadequate urine collection (*n* = 183). This exclusion had only marginal effects on the sizes of associations affecting all-cause mortality. The associations remained between the lowest sex-specific urinary potassium excretion quintile (**Supplemental Table 6** and **Supplemental Figure 2**). Baseline BMI and waist circumference still modified the associations between urinary potassium and mortality (all *P*-interaction < 0.05), in line with the results of the main analyses (**Supplemental Table 7** and **Supplemental Figure 3**A and B). Furthermore, when participants with a history of CVD, malignancy, incident chronic kidney disease, cardiovascular event, or malignancy were excluded, low urinary potassium excretion remained associated with all-cause mortality (HR: 1.71; 95% CI: 1.16; 2.52), **Supplemental Table 8**). Similar results were obtained when participants were excluded who died or were lost to follow-up at ≤1 y after baseline assessment (**Supplemental Table 9**). Finally, additional adjustment for use of RAAS inhibitors or diuretics during follow-up or exclusion of participants using RAAS inhibitors or diuretics during follow-up did not materially change the results (**Supplemental Table 10**).

## Discussion

In this study, we found a reverse J-shaped association between urinary potassium excretion and all-cause mortality in a large, well-characterized, general population-based cohort with 18.4 y of follow-up. Additionally, we found significant effect modification associated with body dimensions.

Prior studies showed effect modification by BMI for the association between urinary potassium excretion and incident hypertension (10). Furthermore, a larger waist circumference was associated with a stronger blood pressure–lowering effect of potassium supplementation (9). In the current study, we found that this higher potassium intake was associated with a higher mortality risk in individuals with obesity. A possible explanation is that urinary potassium excretion correlated with urinary sodium excretion, individuals in the highest potassium quintile excreted higher amounts of sodium than those in lower quintiles, and nearly all individuals with obesity and with high potassium excretion had concomitant sodium intake >120 mmol/24 h (18). Therefore, concurrent high sodium intake could have blunted the inverse association between urinary potassium excretion and mortality in participants with obesity, despite adjustment for urinary sodium excretion. In line with our findings, another observational study found that the association of high potassium intake with lower risks of cardiovascular events and death was blunted by concomitant high sodium intake (19). Alternatively, other unhealthy lifestyle–related factors could also have confounded our results. Our results are in contrast with findings from the NHANES3, where the inverse association between potassium intake by 24-h dietary recall and all-cause mortality was significant in overweight participants, but not in non-overweight participants (20). Other studies, including the more recent PURE (Prospective Urban Rural Epidemiology) study, found no interaction by BMI for the association between urinary potassium excretion and blood pressure (21), all-cause mortality, or CVD (2). Discrepancies between our results and previous findings might be explained by differences in study population ethnicity, study era, assessment of potassium intake (dietary recall, spot urines or 24-h urines), BMI cutoffs, study outcomes, and concomitant sodium intake. Our results set the stage for further studies on the impact of increasing potassium intake in individuals with obesity, also given the increasing prevalence of obesity around the world (22).

Our finding that in the full cohort low potassium intake was associated with an increased mortality risk is in line with several previous studies (1–3). In these studies, the risk of all-cause mortality increased if potassium intake was <2.1–2.5 g/d. In line with this finding, we found an independent relation between a low 24-h urinary potassium excretion (<2.7 g/d) and a higher risk for all-cause mortality in the general population, which supports the WHO recommendation of potassium intake of at least 3.5 g/d (23). Further analyses of cause-specific mortality outcomes showed that urinary potassium excretion was associated with both CVD-related and non–CVD-related mortality. A trend was found in the association with CVD mortality, probably due to a smaller number of events, compared with non–CVD-related mortality. It has been reported that low potassium intake may increase sodium reabsorption, resulting in a higher blood pressure (24). Moreover, endothelial and vascular function are disturbed by low potassium intake, and this effect may be reversed by potassium supplementation (25). Finally, low urinary potassium excretion might reflect lower overall nutritional intake (26), and malnourishment might drive the increased mortality risk. On the other hand, a recent trial in >20,000 individuals at cardiovascular risk showed that a salt substitute that partly replaced sodium with potassium salt lowered the risk of mortality and cardiovascular events (5). These findings suggest that potassium supplementation has specific beneficial effects, although whether the outcomes were driven by potassium supplementation or sodium restriction remains unclear.

Our study has several strengths and limitations. We performed this study in a large and well-characterized cohort with a median follow-up of >18 y. Although no repeated 24-h urinary measurements were available during follow-up (27), 2 independent 24-h urine collections at baseline were used to estimate potassium intake. Findings from our cohort, which consisted predominantly of individuals of European ancestry, may not be generalizable to other populations (28). Our findings are observational, and therefore, residual confounding might persist despite multivariable adjustment. Our study focused on potassium and sodium as individual factors and lacked data on dietary contents, such as fruit and vegetable intake. Some dietary patterns high in potassium, such as the Dietary Approach to Stop Hypertension diet and the Mediterranean diet, also contain other beneficial micronutrients (29,30). Therefore, intake of other nutrients or minerals might have confounded our results.

In conclusion, we found that low urinary potassium excretion was associated with a higher mortality risk in community-dwelling individuals. We found effect modification by body dimensions. In participants with obesity, the association of urinary potassium excretion displayed a U-shaped curve, whereas in participants without obesity this association showed a reverse J-shaped curve. The higher mortality risk in individuals with obesity and with high urinary potassium excretion might be explained by concomitant high sodium intake or other confounding lifestyle-related factors. Our findings set the stage for prospective studies addressing the impact of increasing potassium intake on clinical outcomes in individuals with obesity.

## Supplementary Material

nqac137_Supplemental_File

## Data Availability

Data described in the manuscript, code book, and analytic code will be made available upon request pending.

## Notes

Supported by the Dutch Kidney Foundation (K^+^onsortium, grant CP1601). RAdB is supported by grants from the Netherlands Heart Foundation (CVON SHE-PREDICTS-HF, grant 2017-21; CVON RED-CVD, grant 2017-11; CVON PREDICT2, grant 2018-30; and CVON DOUBLE DOSE, grant 2020B005), by a grant from the leDucq Foundation [Cure PhosphoLambaN induced Cardiomyopathy (Cure-PLaN)], and by a grant from the European Research Council (ERC CoG 818715, SECRETE-HF).

Supplemental Figures 1–3 and Supplemental Tables 1–10 are available from the “Supplementary data” link in the online posting of the article at https://academic.oup.com/ajcn/.

Abbreviations used: CVD, cardiovascular disease; eGFR, estimated glomerular filtration rate; PREVEND, Prevention of Renal and Vascular End-Stage Disease; RAAS, renin-angiotensin-aldosterone system
