# Supplementary material for: Urinary potassium excretion and mortality risk in community-dwelling individuals with and without obesity
Source: Am J Clin Nutr. 2022 May 17;116(3):741–9. doi: 10.1093/ajcn/nqac137 (PMC9437991; doi:10.1093/ajcn/nqac137)
Supplement: nqac137_Supplemental_File [file nqac137_supplemental_file.zip › Supplemental Figures 1-3_FINAL.pptx]

## Slide 1
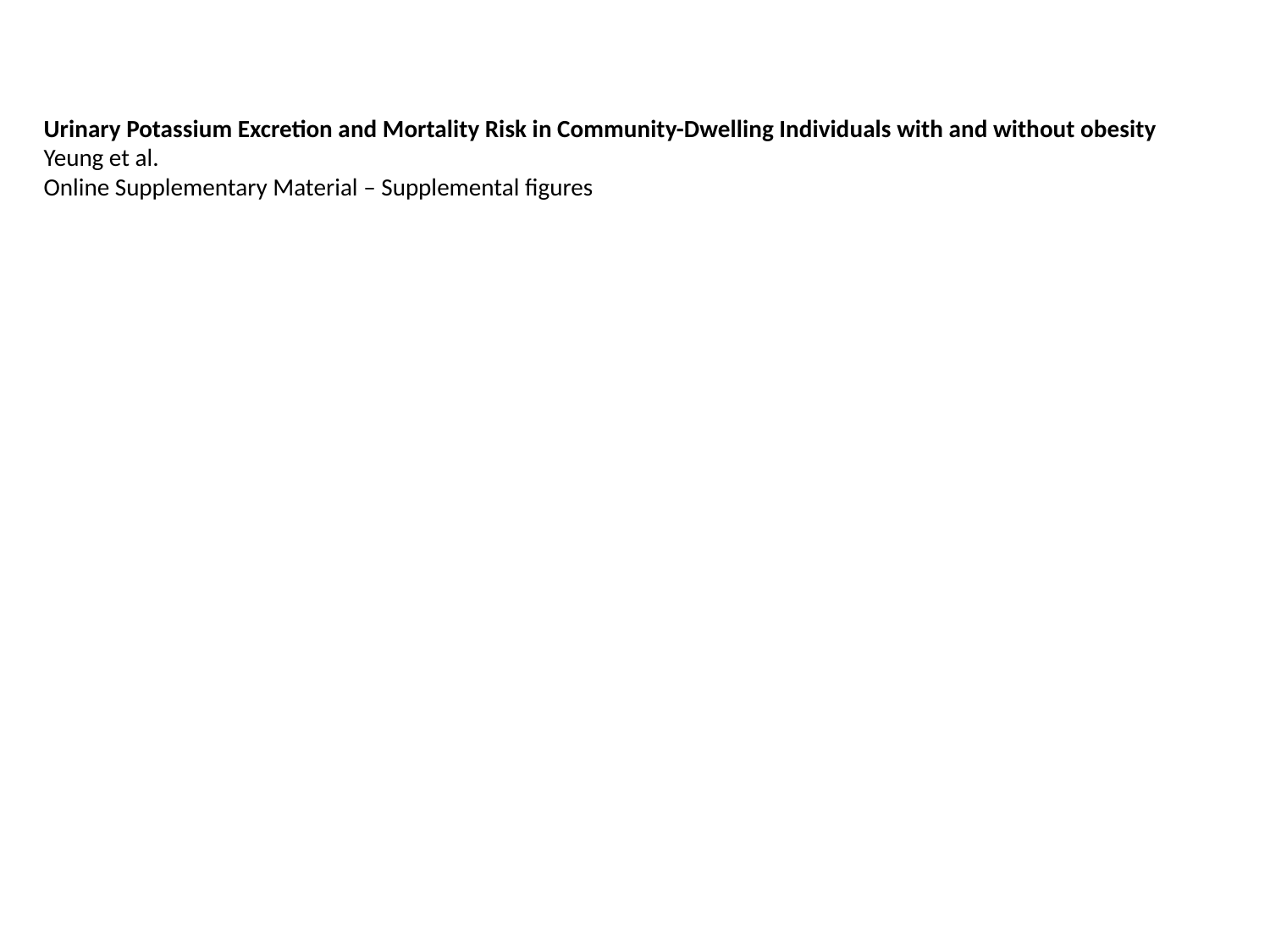

Urinary Potassium Excretion and Mortality Risk in Community-Dwelling Individuals with and without obesity
Yeung et al.
Online Supplementary Material – Supplemental figures

## Slide 2
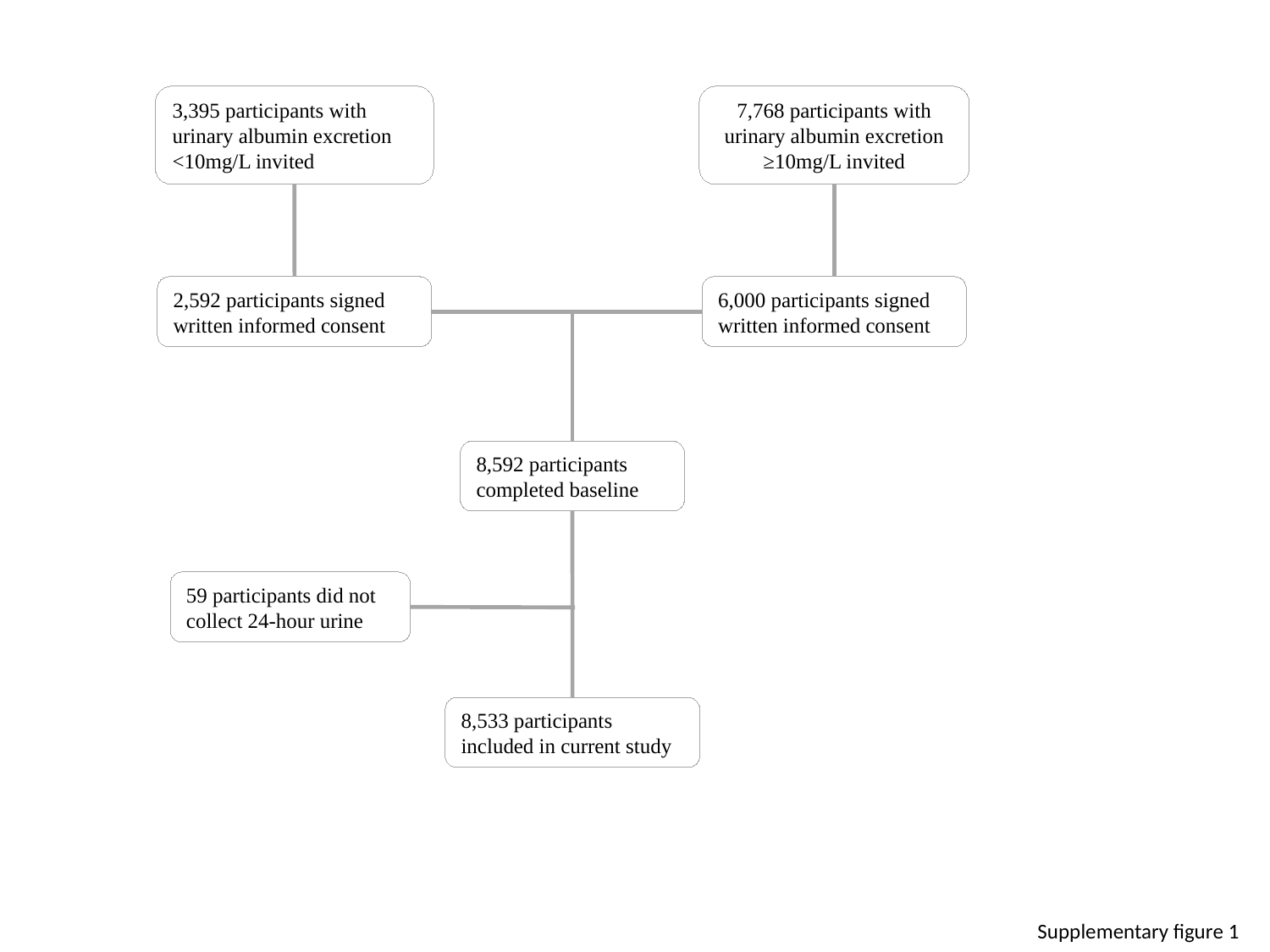

3,395 participants with urinary albumin excretion <10mg/L invited
7,768 participants with urinary albumin excretion ≥10mg/L invited
2,592 participants signed written informed consent
6,000 participants signed written informed consent
8,592 participants completed baseline
59 participants did not collect 24-hour urine
8,533 participants included in current study
Supplementary figure 1

## Slide 3
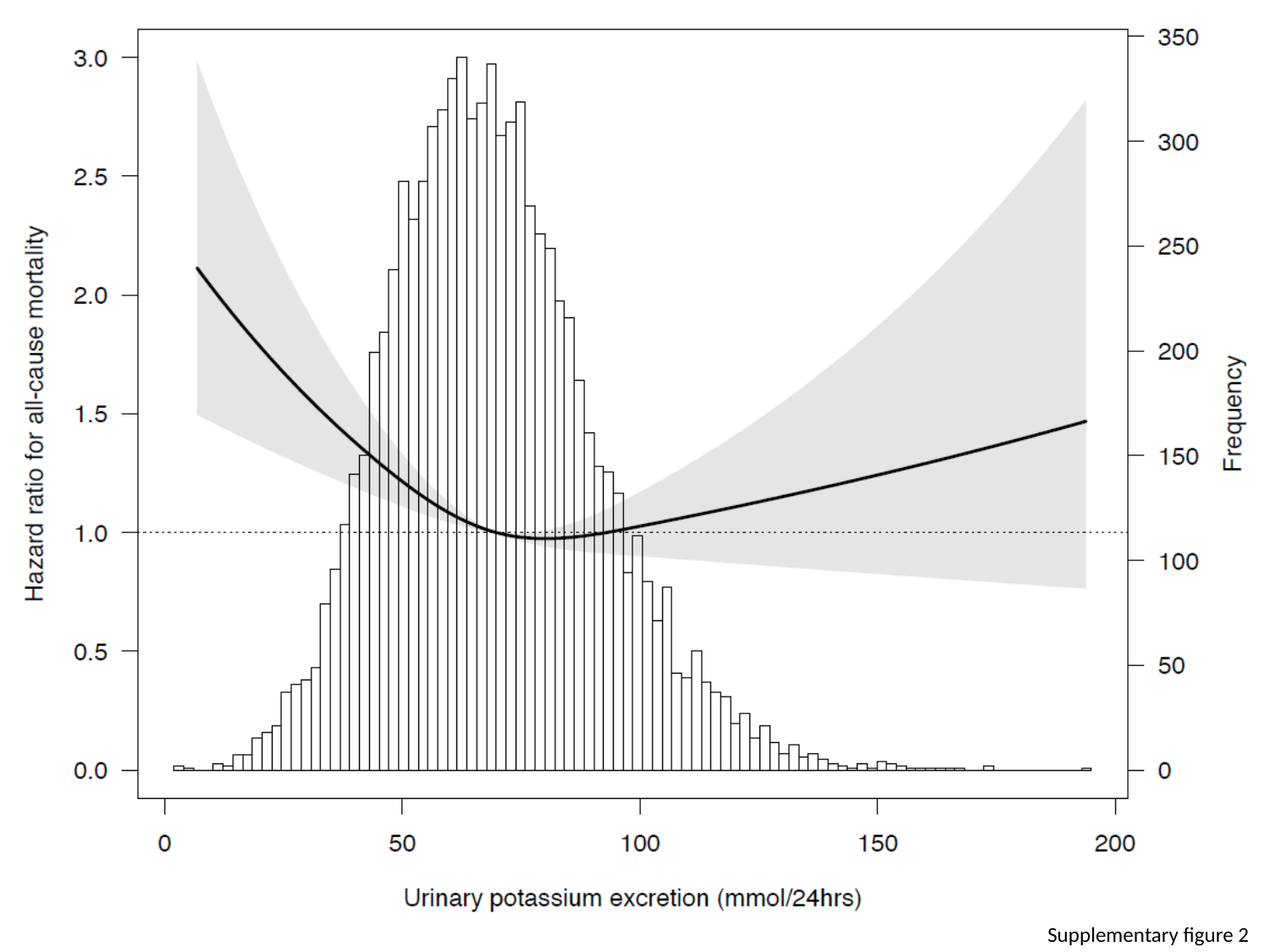

#
Supplementary figure 2

## Slide 4
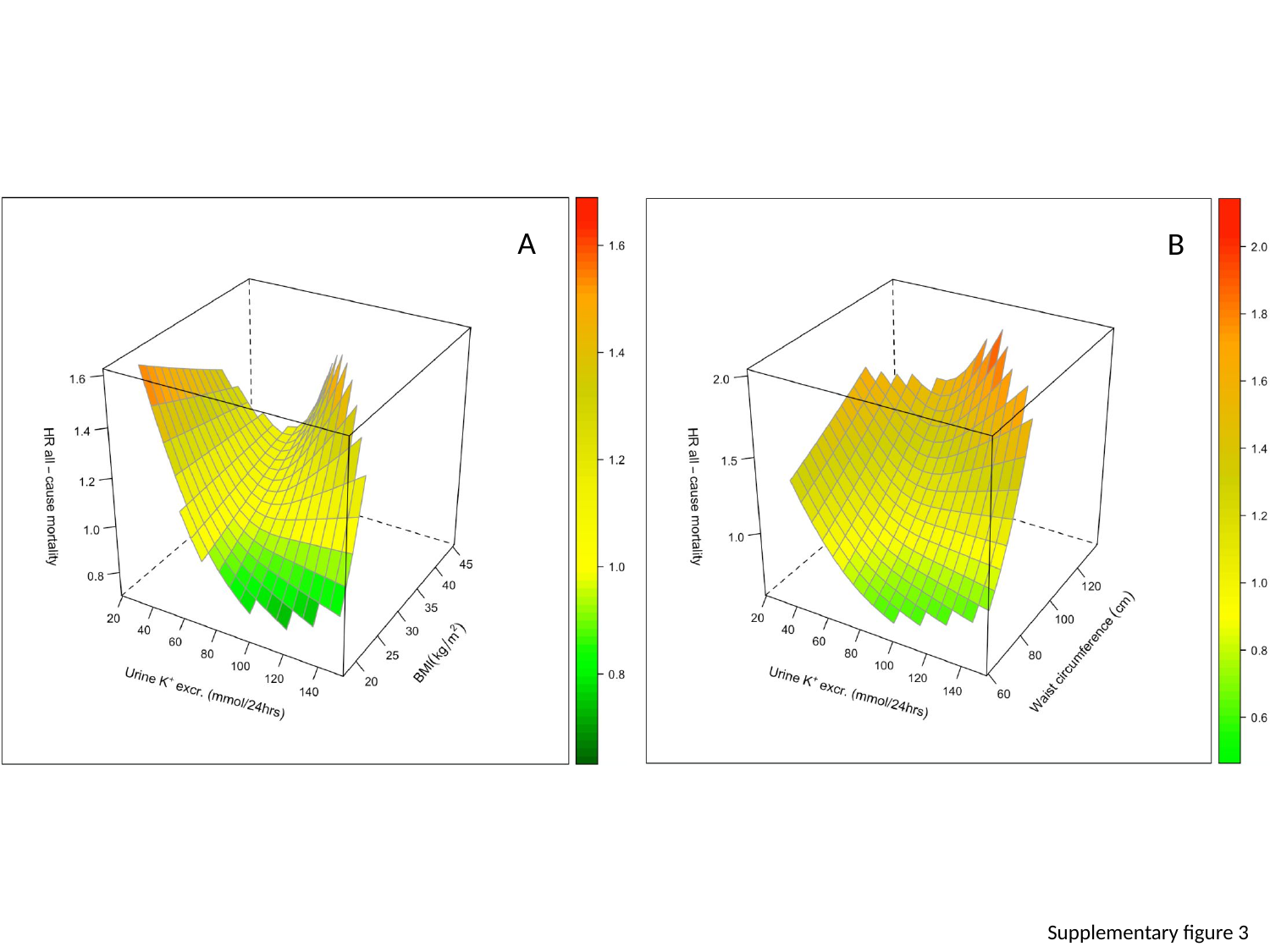

A
B
Supplementary figure 3
