# Supplementary material for: Urinary potassium excretion and mortality risk in community-dwelling individuals with and without obesity
Source: Am J Clin Nutr. 2022 May 17;116(3):741–9. doi: 10.1093/ajcn/nqac137 (PMC9437991; doi:10.1093/ajcn/nqac137)
Supplement: nqac137_Supplemental_File [file nqac137_supplemental_file.zip › Supplemental Files_final.docx]

# Urinary Potassium Excretion and Mortality Risk Community-Dwelling Individuals with and without obesity

Stanley M.H. Yeung^1^*, Anne Nooteboom^1^*, Ewout J. Hoorn^2^, Joris I. Rotmans^3^, Liffert Vogt^4^, Rudolf A. de Boer^5^, Ron T. Gansevoort^1^, Gerjan Navis^1^, Stephan J. L. Bakker^1^, Martin H. De Borst^1^

## Online Supplementary Material

^1^Department of Internal Medicine, Division of Nephrology, University of Groningen, University Medical Center Groningen, Groningen, the Netherlands

^2^Department of Internal Medicine, Division of Nephrology & Transplantation, Erasmus Medical Center, University Medical Center Rotterdam, Rotterdam, The Netherlands

^3^Department of Internal Medicine, Leiden University Medical Center, Leiden, The Netherlands

^4^Department of Internal Medicine, Section of Nephrology, Amsterdam Cardiovascular Sciences, Amsterdam University Medical Centers, University of Amsterdam, Amsterdam, The Netherlands

^5^Department of Cardiology, University of Groningen, University Medical Center Groningen, Groningen, the Netherlands

Short title: Urine potassium and mortality in obese individuals

Corresponding author:

Stanley M.H. Yeung, MD

Email: m.h.yeung@umcg.nl

Department of Internal Medicine, Division of Nephrology

University of Groningen, University Medical Center Groningen

Hanzeplein 1, P.O. Box 30.001, 9700 RB Groningen, The Netherlands

Tel: +31 50 361 5199, Fax: +31 50 361 9350

## Online Supplementary Material

## Table of contents

**Legends for supplemental figures**

**Supplemental Figure 1** Flow diagram of participant inclusion

**Supplemental Figure 2** Associations between urinary potassium excretion and all-cause mortality in 8,350 participants after exclusion of potentially inadequate urine collections.

**Supplemental Figure 3** 3D plots depicting the multivariable adjusted association between urinary potassium excretion (BMI, A; Waist circumference, B), and all-cause mortality in 8,350 participants after exclusion of potentially inadequate urine collections.

**Supplemental Tables**

**Supplemental Table 1** Number of participants with missing data for each variable

**Supplemental Table 2.** Association of urinary potassium excretion with risk of all-cause mortality with additional adjustment for potential mediators in 8,533 subjects of PREVEND

**Supplemental Table 3.** Association of urinary potassium excretion with risk of CVD and non-CVD mortality in 8,533 subjects of PREVEND

**Supplemental Table 4.** Association of urinary potassium excretion with all-cause mortality with additional adjustment for potential mediators in individuals with a BMI ≥30 kg/m^2^ or waist circumference ≥94cm in 1,333 subjects of PREVEND

**Supplemental Table 5.** Association between urinary sodium excretion and sex-specific quintiles of urinary potassium excretion in 1,333 obese (BMI ≥30kg/m^2^) participants of PREVEND

**Supplemental Table 6.** Association of urinary potassium excretion with risk of all-cause mortality in 8,350 subjects of PREVEND (after exclusion of potentially inadequate urine collections)

**Supplemental Table 7.** Association of urinary potassium excretion with all-cause mortality in body composition subgroups (after exclusion of potentially inadequate urine collections)

**Supplemental Table 8.** Association of urinary potassium excretion with risk of all-cause mortality in 4,871 PREVEND participants (without history of CVD or malignancy and without participants with incident kidney progression, cardiovascular event, or malignancy)

**Supplemental Table 9.** Association of urinary potassium excretion with risk of all-cause mortality in 8,477 PREVEND participants (with a follow-up ≥1 year)

**Supplemental Table 10.** Association of urinary potassium excretion with risk of all-cause mortality in 8,533 PREVEND participants and in participants without use of RAAS-inhibitors or diuretics in the follow-up (6,767 participants)

## Legends for supplemental figures

**Supplemental Figure 1** Flow diagram of participant inclusion

**Supplemental Figure 2** Associations between urinary potassium excretion and all-cause mortality in 8,350 participants without potential inadequate urine collection. Data were fit by a Cox proportional hazards regression model based on restricted cubic splines (5^th^, 50^th^, and 95^th^ percentile knots) and adjusted for age, sex, eGFR, BMI, urinary albumin-to-creatinine excretion, type 2 diabetes, education level, alcohol consumption, smoking, history of cardiovascular disease, triglyceride HDL ratio, urinary creatinine excretion, urea excretion, sodium excretion, antihypertensive, anti-diabetic and lipid lowering drugs. The grey area represents the 95% CI. Abbreviations: HR, hazard ratio, eGFR, estimated glomerular filtration rate; BMI; body mass index.

**Supplemental Figure 3** 3D plots depicting the multivariable adjusted association between urinary potassium excretion (BMI, A; Waist circumference, B), and all-cause mortality in 8,350 participants without potential inadequate urine collection. Cox proportional hazards regression model was adjusted for age, sex, eGFR, BMI, urinary albumin-to-creatinine excretion, type 2 diabetes, education level, alcohol consumption, smoking, history of cardiovascular disease, triglyceride HDL ratio, urinary creatinine excretion, urea excretion, sodium excretion, antihypertensive, anti-diabetic and lipid lowering drugs. Abbreviations: HR, hazard ratio, eGFR, estimated glomerular filtration rate; BMI; body mass index.

**Supplemental Table 1.** Number of participants with missing data for each variable

| Variable | Number of missing (%) |
| --- | --- |
| BMI | 91 (1.1) |
| Waist circumference | 93 (1.1) |
| Systolic blood pressure | 4 (0.0) |
| Diastolic blood pressure | 4 (0.0) |
| Antihypertensive drugs | 263 (3.1) |
| ACE-inhibitors/ARB | 1511 (17.7) |
| Thiazide diuretics | 1276 (15.0) |
| Loop diuretics | 1278 (15.0) |
| Potassium sparing diuretics | 1277 (15.0) |
| Lipid lowering drugs | 1511 (17.7) |
| Oral hypoglycemic drugs | 1511 (17.7) |
| Education | 42 (0.5) |
| Smoking status | 31 (0.4) |
| Alcohol consumption | 43 (0.5) |
| Type 2 Diabetes | 122 (1.4) |
| History of malignancy | 81 (0.9) |
| Plasma creatinine | 566 (6.6) |
| eGFR (CKD-epi) | 571 (6.7) |
| Plasma albumin | 1160 (13.6) |
| Plasma potassium | 1159 (13.6) |
| Plasma sodium | 1159 (13.6) |
| Serum aldosterone | 1036 (12.1) |
| Plasma renin | 300 (3.5) |
| Total cholesterol | 69 (0.8) |
| HDL cholesterol | 209 (2.4) |
| Triglycerides | 208 (2.4) |
| Triglyceride/HDL ratio | 209 (2.4) |
| Urinary creatinine excretion | 45 (0.5) |
| ACR | 108 (1.3) |

Abbreviations: BMI, body mass index; eGFR, estimated glomerular filtration rate; ACR, albumin-to-creatinine ratio; HDL, high density lipoprotein.

**Supplemental Table 2.** Association of urinary potassium excretion with risk of all-cause mortality with additional adjustment for potential mediators in 8,533 subjects of PREVEND

|  |  | Hazard ratio (95% CI) | | | | |
| --- | --- | --- | --- | --- | --- | --- |
| Sex-specific quintiles of  urinary potassium excretion, mmol/24hrs | Male | < 59 | 59 – 70 | 70 – 81 | 81 – 94 | > 94 |
|  | Female | < 50 | 50 – 60 | 60 – 69 | 69 – 81 | > 81 |
| Fully adjusted HR^1^ |  | 1.38 (1.18, 1.60)^***^ | 1.13 (0.97, 1.32) | 1.0 (ref.) | 0.99 (0.84, 1.16) | 1.01 (0.85, 1.21) |
| Fully adjusted^1^ + serum aldosterone |  | 1.38 (1.18, 1.60)^***^ | 1.13 (0.97, 1.32) | 1.0 (ref.) | 0.99 (0.84, 1.16) | 1.02 (0.85, 1.22) |
| Fully adjusted ^1^+ plasma renin |  | 1.38 (1.19, 1.61)^***^ | 1.13 (0.97, 1.32) | 1.0 (ref.) | 0.98 (0.84, 1.16) | 1.02 (0.85, 1.22) |
| Fully adjusted ^1^+ systolic blood pressure |  | 1.38 (1.18, 1.60)^***^ | 1.13 (0.97, 1.32) | 1.0 (ref.) | 0.99 (0.84, 1.16) | 1.02 (0.85, 1.22) |
| Fully adjusted ^1^+ diastolic blood pressure |  | 1.37 (1.18, 1.60)^***^ | 1.13 (0.97, 1.32) | 1.0 (ref.) | 1.00 (0.85, 1.17) | 1.02 (0.85, 1.22) |

Data are presented as hazard ratio (95% confidence interval); P-value is shown as: * ≤0.05, ** ≤ 0.01, *** <0.001. Abbreviations: CI, confidence interval; HR, hazard ratio; BMI, body mass index; eGFR, estimated glomerular filtration rate; ACR, albumin-to-creatinine ratio; HDL, high density lipoprotein.

^1^Model additionally adjusted for BMI, eGFR, urinary ACR, type 2 diabetes, education level, alcohol consumption, smoking, history of cardiovascular disease, triglyceride HDL ratio, urinary creatinine, urea excretion, antihypertensive, anti-diabetic and lipid lowering drugs.

**Supplemental Table 3.** Association of urinary potassium excretion with risk of CVD and non-CVD mortality in 8,533 subjects of PREVEND

|  |  | Hazard ratio (95% CI) | | | | |
| --- | --- | --- | --- | --- | --- | --- |
| Sex-specific quintiles of  urinary potassium excretion, mmol/24hrs | Male | < 59 | 59 – 70 | 70 – 81 | 81 – 94 | > 94 |
|  | Female | < 50 | 50 – 59 | 59 – 69 | 69 – 81 | > 81 |
| Cardiovascular mortality |  |  |  |  |  |  |
| HR adjusted for age |  | 1.35 (1.02, 1.77)^*^ | 1.13 (0.85, 1.51) | 1.0 (ref.) | 0.94 (0.69, 1.28) | 0.97 (0.69, 1.37) |
| Fully adjusted HR^1^ |  | 1.33 (0.99, 1.78) | 1.17 (0.87, 1.57) | 1.0 (ref.) | 1.01 (0.74, 1.37) | 1.00 (0.70, 1.42) |
| Non-cardiovascular mortality |  |  |  |  |  |  |
| HR adjusted for age |  | 1.46 (1.24, 1.73)^***^ | 1.13 (0.94, 1.35) | 1.0 (ref.) | 0.93 (0.77, 1.12) | 0.99 (0.81, 1.20) |
| Fully adjusted HR^1^ |  | 1.40 (1.17, 1.68)^***^ | 1.13 (0.94, 1.35) | 1.0 (ref.) | 0.96 (0.80, 1.17) | 1.02 (0.83, 1.26) |

Data are presented as hazard ratio (95% confidence interval); P-value is shown as: * ≤0.05, ** ≤ 0.01, *** <0.001. Abbreviations: CVD, cardiovascular disease; CI, confidence interval; HR, hazard ratio; BMI, body mass index; eGFR, estimated glomerular filtration rate; ACR, albumin-to-creatinine ratio; HDL, high density lipoprotein.

^1^Model additionally adjusted for BMI, eGFR, urinary ACR, type 2 diabetes, education level, alcohol consumption, smoking, history of cardiovascular disease, triglyceride HDL ratio, urinary creatinine, urea excretion, antihypertensive, oral hypoglycemic and lipid lowering drugs.

**Supplemental Table 4.** Association of urinary potassium excretion with all-cause mortality with additional adjustment for potential mediators in individuals with a BMI ≥30 kg/m^2^ or waist circumference ≥94cm in 1,333 subjects of PREVEND

|  | Sex-specific quintiles of urinary potassium excretion (mmol/24hrs) Hazard ratio (95% CI) | | | | |
| --- | --- | --- | --- | --- | --- |
|  | **I** | **II** | **III** | **IV** | **V** |
| **BMI ≥30 kg/m^2^** |  |  |  |  |  |
| MA | 1.47 (1.00, 2.16)^*^ | 1.35 (0.90, 2.02) | 1.19 (0.79, 1.78) | 1.11 (0.74, 1.67) | 1.52 (1.00, 2.30)^*^ |
| MA + serum aldosterone | 1.48 (1.01, 2.18)^*^ | 1.36 (0.91, 2.03) | 1.19 (0.80, 1.79) | 1.12 (0.74, 1.68) | 1.53 (1.01, 2.31)^*^ |
| MA + plasma renin | 1.45 (0.99, 2.14) | 1.36 (0.91, 2.03) | 1.19 (0.80, 1.79) | 1.12 (0.74, 1.68) | 1.54 (1.02, 2.33)^*^ |
| MA + systolic blood pressure | 1.47 (1.00, 2.16)^*^ | 1.33 (0.89, 1.99) | 1.18 (0.79, 1.78) | 1.11 (0.74, 1.67) | 1.51 (1.00, 2.28) |
| MA + diastolic blood pressure | 1.44 (0.98, 2.12) | 1.32 (0.88, 1.97) | 1.19 (0.80, 1.79) | 1.11 (0.74, 1.68) | 1.51 (1.00, 2.29) |
| **Waist circumference ≥94cm** |  |  |  |  |  |
| MA | 1.50 (1.16, 1.94)^**^ | 1.45 (1.13, 1.90)^**^ | 1.35 (1.04, 1.76)^*^ | 1.14 (0.86, 1.50) | 1.36 (1.02, 1.80)^*^ |
| MA + serum aldosterone | 1.50 (1.16, 1.94)^**^ | 1.46 (1.13, 1.90)^**^ | 1.35 (1.04, 1.76)^*^ | 1.14 (0.86, 1.50) | 1.36 (1.02, 1.81)^*^ |
| MA + plasma renin | 1.50 (1.16, 1.94)^**^ | 1.45 (1.12, 1.88)^**^ | 1.35 (1.04, 1.76)^*^ | 1.13 (0.86, 1.50) | 1.38 (1.04, 1.83)^*^ |
| MA + systolic blood pressure | 1.49 (1.15, 1.93)^**^ | 1.45 (1.12, 1.88)^**^ | 1.34 (1.03, 1.75)^*^ | 1.13 (0.86, 1.49) | 1.35 (1.02, 1.80)^*^ |
| MA + diastolic blood pressure | 1.47 (1.13, 1.90)^**^ | 1.44 (1.11, 1.87)^**^ | 1.33 (1.02, 1.73)^*^ | 1.13 (0.86, 1.49) | 1.34 (1.01, 1.78)^*^ |

Multivariable adjusted Cox regression models (MA) adjusted for age, BMI, eGFR, urinary ACR, type 2 diabetes, education level, alcohol consumption, smoking, history of cardiovascular disease, triglyceride HDL ratio, urinary creatinine, urea, and sodium excretion, antihypertensive, anti-diabetic and lipid lowering drugs. Reference group for BMI was third quintile of urinary potassium excretion and BMI <25 kg/m^2^, for waist circumference was third quintile of urinary potassium excretion and waist circumference 82–94 cm. Subsequently, serum (Ln) aldosterone, plasma (Ln) renin, systolic and diastolic blood pressure were individually added to the MA model. Data are presented as HR and 95% CI; Abbreviations: HR, hazard ratio; 95% CI, confidence interval; LN, Natural logarithm; BMI, Body mass index; eGFR, estimated glomerular filtration rate; ACR, albumin-to-creatinine ratio; HDL, high density lipoprotein; P-value is shown as: * ≤0.05, ** ≤ 0.01.

**Supplemental Table 5.** Association between urinary sodium excretion and sex-specific quintiles of urinary potassium excretion in 1,333 obese (BMI ≥30kg/m^2^) participants of PREVEND

| **Sex-specific quintiles of total urinary potassium excretion, mmol/24-h** | | | | | | | |
| --- | --- | --- | --- | --- | --- | --- | --- |
|  | Total | **I** | **II** | **III** | **IV** | **V** | **P-trend*** |
| Participants, n | 1,333 | 266 | 267 | 267 | 268 | 265 |  |
| Male | 80 ± 22 | < 61 | 61 – 74 | 74 – 85 | 85 – 97 | > 97 |  |
| Female | 66 ± 20 | < 50 | 50 - 60 | 60 - 69 | 69 – 82 | > 82 |  |
| Urinary sodium excretion, (mmol/24hrs) | 156 (120–197) | 119 (84–155) | 141 (116–171) | 162 (127–203) | 170 (135–209) | 187 (157–230) | <0.001 |
| Participants with urinary sodium excretion <120mmol/24hrs, n (%) | 335 (25) | 136 (51) | 79 (30) | 56 (21) | 41 (15) | 23 (9) | <0.001 |

**Supplemental Table 6.** Association of urinary potassium excretion with risk of all-cause mortality in 8,350 subjects of PREVEND (without potential inadequate urine collection)

|  |  | Hazard ratio (95% CI) | | | | |
| --- | --- | --- | --- | --- | --- | --- |
| Sex-specific quintiles of  urinary potassium excretion, mmol/24hrs | Male | < 59 | 59 – 70 | 70 – 81 | 81 – 94 | > 94 |
|  | Female | < 50 | 50 – 60 | 60 – 69 | 69 – 81 | > 81 |
| HR adjusted for age |  | 1.40 (1.21, 1.62)^***^ | 1.15 (0.98, 1.34) | 1.0 (ref.) | 0.95 (0.81, 1.12) | 1.00 (0.84, 1.19) |
| Fully adjusted HR^1^ |  | 1.38 (1.18, 1.61)^***^ | 1.16 (0.99, 1.35) | 1.0 (ref.) | 0.99 (0.84, 1.17) | 1.02 (0.85, 1.22) |

Data are presented as hazard ratio (95% confidence interval); P-value is shown as: * ≤0.05, ** ≤ 0.01, *** <0.001. Abbreviations: CI, confidence interval; HR, hazard ratio; BMI, body mass index; eGFR, estimated glomerular filtration rate; ACR, albumin-to-creatinine ratio; HDL, high density lipoprotein.

^1^Model additionally adjusted for BMI, eGFR, urinary ACR, type 2 diabetes, education level, alcohol consumption, smoking, history of cardiovascular disease, triglyceride HDL ratio, urinary sodium, creatinine, urea excretion, antihypertensive, anti-diabetic and lipid lowering drugs.

**Supplemental Table 7.** Association of urinary potassium excretion with all-cause mortality in body composition subgroups (without potential inadequate urine collection)

|  | Sex-specific quintiles of urinary potassium excretion (mmol/24hrs) Hazard ratio (95% CI) | | | | |  |
| --- | --- | --- | --- | --- | --- | --- |
|  | **I** | **II** | **III** | **IV** | **V** | ***P*-interaction** |
| BMI <25 kg/m^2^ | 160/563 | 100/723 | 87/724 | 77/725 | 44/723 | <0.001 |
|  | 1.72 (1.32, 2.25)^***^ | 1.13 (0.85, 1.51) | 1.0 (ref.) | 1.18 (0.87, 1.61) | 0.89 (0.61, 1.28) |  |
| BMI 25–30 kg/m^2^ | 222/686 | 192/688 | 147/687 | 129/688 | 107/687 |  |
|  | 1.37 (1.04, 1.81)^*^ | 1.36 (1.03, 1.79)^*^ | 1.09 (0.82, 1.46) | 0.88 (0.66, 1.19) | 1.14 (0.84, 1.55) |  |
| BMI ≥30 kg/m^2^ | 81/258 | 71/260 | 66/259 | 61/260 | 62/259 |  |
|  | 1.44 (0.97, 2.13) | 1.44 (0.97, 2.13) | 1.22 (0.81, 1.85) | 1.14 (0.76, 1.73) | 1.50 (0.99, 2.29) |  |
| Waist circumference <82 cm | 74/527 | 41/528 | 38/529 | 34/527 | 22/527 | 0.001 |
|  | 1.50 (1.08, 2.08)^*^ | 0.92 (0.62, 1.34) | 0.79 (0.54, 1.17) | 1.00 (0.67, 1.51) | 0.83 (0.52, 1.34) |  |
| Waist circumference 82–94 cm | 159/570 | 112/569 | 88/571 | 87/569 | 52/570 |  |
|  | 1.54 (1.17, 2.01)^*^ | 1.22 (0.92, 1.62) | 1.0 (ref.) | 1.20 (0.78, 1.62) | 0.90 (0.63, 1.27) |  |
| Waist circumference ≥94 cm | 230/572 | 202/572 | 184/574 | 145/574 | 138/571 |  |
|  | 1.53 (1.19, 1.98)^**^ | 1.53 (1.18, 1.98)^**^ | 1.37 (1.06, 1.79)^*^ | 1.15 (0.87, 1.52) | 1.42 (1.06, 1.88)^*^ |  |

Multivariable adjusted Cox regression models adjusted for age, BMI, eGFR, urinary ACR, type 2 diabetes, education level, alcohol consumption, smoking, history of cardiovascular disease, triglyceride HDL ratio, urinary creatinine, urea, and sodium excretion, antihypertensive, anti-diabetic and lipid lowering drugs. Data are presented as HR, hazard ratio; 95% CI, confidence interval; P-value is shown as: * ≤0.05, ** ≤ 0.01, *** <0.001. Abbreviations: BMI, Body mass index; eGFR, estimated glomerular filtration rate; ACR, albumin-to-creatinine ratio; HDL, high density lipoprotein; N, number of participants; n, number of events.

**Supplemental Table 8.** Association of urinary potassium excretion with risk of all-cause mortality in 4,871 PREVEND participants without history of CVD or malignancy and without participants with incident chronic kidney disease, cardiovascular event, or malignancy during follow-up

|  |  | Hazard ratio (95% CI) | | | | |
| --- | --- | --- | --- | --- | --- | --- |
| Sex-specific quintiles of  urinary potassium excretion, mmol/24hrs | Male | < 61 | 61 – 73 | 73 – 83 | 83 – 96 | > 96 |
|  | Female | < 50 | 50 – 60 | 60 – 70 | 70 – 81 | > 81 |
| HR adjusted for age |  | 2.19 (1.55, 3.11)^***^ | 1.16 (0.78, 1.72) | 1.0 (ref.) | 1.56 (1.06, 2.29)^*^ | 1.30 (0.86, 1.98) |
| Fully adjusted HR^1^ |  | 1.73 (1.19, 2.52)^**^ | 1.13 (0.76, 1.69) | 1.0 (ref.) | 1.76 (1.20, 2.60)^**^ | 1.56 (1.01, 2.40)^*^ |

Data are presented as hazard ratio (95% confidence interval); P-value is shown as: * ≤0.05, ** ≤ 0.01, *** <0.001. Abbreviations: CVD, cardiovascular disease; CI, confidence interval; HR, hazard ratio; BMI, body mass index; eGFR, estimated glomerular filtration rate; ACR, albumin-to-creatinine ratio; HDL, high density lipoprotein.

^1^Model additionally adjusted for BMI, eGFR, urinary ACR, type 2 diabetes, education level, alcohol consumption, smoking, history of cardiovascular disease, triglyceride HDL ratio, urinary creatinine, urea excretion, antihypertensive, oral hypoglycemic and lipid lowering drugs.

**Supplemental Table 9.** Association of urinary potassium excretion with risk of all-cause mortality in 8,477 PREVEND participants (with a follow-up ≥1 year)

|  |  | Hazard ratio (95% CI) | | | | |
| --- | --- | --- | --- | --- | --- | --- |
| Sex-specific quintiles of  urinary potassium excretion, mmol/24hrs | Male | < 59 | 59 – 70 | 70 – 81 | 81 – 94 | > 94 |
|  | Female | < 50 | 50 – 60 | 60 – 69 | 69 – 81 | > 81 |
| HR adjusted for age |  | 1.44 (1.22, 1.71)^***^ | 1.11 (0.93, 1.33) | 1.0 (ref.) | 0.93 (0.76, 1.12) | 0.97 (0.79, 1.19) |
| Fully adjusted HR^1^ |  | 1.38 (1.15, 1.65)^**^ | 1.10 (0.92, 1.32) | 1.0 (ref.) | 0.96 (0.79, 1.16) | 1.00 (0.81, 1.24) |

Data are presented as hazard ratio (95% confidence interval); P-value is shown as: * ≤0.05, ** ≤ 0.01, *** <0.001. Abbreviations: CVD, cardiovascular disease; CI, confidence interval; HR, hazard ratio; BMI, body mass index; eGFR, estimated glomerular filtration rate; ACR, albumin-to-creatinine ratio; HDL, high density lipoprotein.

^1^Model additionally adjusted for BMI, eGFR, urinary ACR, type 2 diabetes, education level, alcohol consumption, smoking, history of cardiovascular disease, triglyceride HDL ratio, urinary creatinine, urea excretion, antihypertensive, oral hypoglycemic and lipid lowering drugs.

**Supplemental Table 10.** Association of urinary potassium excretion with risk of all-cause mortality in 8,533 PREVEND participants and in participants without use of RAAS-inhibitors or diuretics during follow-up (6,767 participants)

|  |  | Hazard ratio (95% CI) | | | | |
| --- | --- | --- | --- | --- | --- | --- |
| Sex-specific quintiles of  urinary potassium excretion, mmol/24hrs | Male | < 59 | 59 – 70 | 70 – 81 | 81 – 94 | > 94 |
|  | Female | < 50 | 50 – 60 | 60 – 69 | 69 – 81 | > 81 |
| Fully adjusted HR^1^ |  | 1.38 (1.15, 1.65)^**^ | 1.10 (0.92, 1.32) | 1.0 (ref.) | 0.96 (0.79, 1.16) | 1.00 (0.81, 1.24) |
| Fully adjusted HR^2^ |  | 1.33 (1.14, 1.55)^***^ | 1.14 (0.98, 1.33) | 1.0 (ref.) | 0.97 (0.82, 1.14) | 1.06 (0.88, 1.26) |
|  |  |  |  |  |  |  |
| *After exclusion of participants using RAAS-inhibitors or diuretics during follow-up (N=6,767 in analysis):* | | | | |  |  |
| Sex-specific quintiles of  urinary potassium excretion, mmol/24hrs | Male | < 59 | 59 – 70 | 70 – 81 | 81 – 94 | > 94 |
|  | Female | < 50 | 50 – 60 | 60 – 69 | 69 – 81 | > 81 |
| HR adjusted for age |  | 1.49 (1.26, 1.77)^***^ | 1.16 (0.97, 1.39) | 1.0 (ref.) | 0.97 (0.80, 1.18) | 0.96 (0.77, 1.18) |
| Fully adjusted HR^1^ |  | 1.43 (1.19, 1.72)^**^ | 1.16 (0.97, 1.40) | 1.0 (ref.) | 0.98 (0.81, 1.19) | 0.99 (0.79, 1.24) |

Data are presented as hazard ratio (95% confidence interval); P-value is shown as: * ≤0.05, ** ≤ 0.01, *** <0.001. Abbreviations: CVD, cardiovascular disease; CI, confidence interval; HR, hazard ratio; BMI, body mass index; eGFR, estimated glomerular filtration rate; ACR, albumin-to-creatinine ratio; HDL, high density lipoprotein.

^1^Model additionally adjusted for BMI, eGFR, urinary ACR, type 2 diabetes, education level, alcohol consumption, smoking, history of cardiovascular disease, triglyceride HDL ratio, urinary creatinine, urea excretion, antihypertensive, oral hypoglycemic and lipid lowering drugs. ^2^Model additionally adjusted for use of RAAS-inhibitors or diuretics during follow up.
